# Transitions to Sociobioeconomy in the Amazon: Governance, Business, and Sustainable Innovations

**DOI:** 10.1007/s00267-026-02532-y

**Published:** 2026-09-03

**Authors:** Maria Sylvia Macchione Saes, Adriana Marotti de Mello, Elis Regina Monte Feitosa, José Augusto Lacerda Fernandes, Zilda Joaquina Cohen Gama dos Santos, Kavita Miadaira Hamza

**Affiliations:** 1 School of Economics, Management, Accounting and Actuarial Sciences, University of São Paulo (USP), São Paulo, Brazil; 2 ICSA, Federal University of Pará (UFPA), Belém, Brazil; 3 Social Sciences Institute, Federal University of Western Pará, Santarem, Brazil

**Keywords:** Sustainable transitions, Polycentric governance, Bioeconomy, Value chain

## Abstract

Sociobioeconomy has gained prominence as an alternative to conventional bioeconomy by integrating biodiversity conservation, social inclusion, and the valorization of traditional knowledge. This article aims to identify the institutional and organizational elements that enable sociotechnical transitions to sociobioeconomy in the Amazon. The study adopts a qualitative and exploratory approach based on the analysis of four initiatives that reconcile nature conservation, social inclusion, and traditional knowledge: the sustainable management of pirarucu, fine cocoa and chocolate production in Transamazônica, the craftsmanship of cuia from Baixo Amazonas (collective brand Aíra), and the Origens Brasil Platform. The results show that successful experiences depend on polycentric governance, community participation, local leadership, multiscale collaborative networks, and equitable value distribution mechanisms. These initiatives show concrete signs of transition, such as ecological recovery, increased local income, productive autonomy, cultural strengthening, and access to differentiated markets, although they face structural barriers related to logistics, difficulties in obtaining certifications, informality, and institutional limitations. Thus, it is evident that transitions to sociobioeconomy require deep transformations in the dominant regime, including the reconfiguration of value chains, strengthening of polycentric arrangements, and the construction of public policies that promote inclusion, social justice, and socio-environmental conservation.

## Introduction

Bioeconomy has been identified as one of the main strategies to promote transition towards sustainability (Garrett et al., 2024). However, the bioeconomy, as adopted in the Global North, which focuses on producing bioinputs to energy transition and using biotechnology in industry, fails to address the complexities of Global South, such as the Amazon region in Brazil (Ferreira et al., 2024). In these countries, sustainable transitions require strategies that transcend forest preservation to include the large population that depends on these resources for their livelihood (Abramovay et al., 2021; Saes et al., 2023).

In this context, sociobioeconomy, which adopts a perspective oriented towards biodiversity conservation and social inclusion with protagonism of local communities, emerges as an alternative to conventional bioeconomy, which often reproduces predatory practices and deepens historical inequalities and power asymmetries (Ferreira et al., 2024; Abramovay et al., 2021; Saes et al., 2023).

The transition from conventional bioeconomy to sociobioeconomy is not trivial. In the Amazon, bioeconomy is commonly characterized by extractive practices, informality, and low value addition (Barbosa et al., 2024; Rosenfeld et al., 2024). The theoretical contributions of Ostrom (1990) become fundamental in demonstrating that sustainable solutions to collective action problems depend on institutions rooted in the territory, with rules adjusted to the local context, legitimized by the actors involved, and sustained by monitoring and conflict resolution mechanisms. This perspective is reinforced by studies showing that sociobioeconomy experiences thrive when embedded in polycentric arrangements combining local collective action, external alliances, and multiscale governance (Brondizio et al., 2021).

Based on this observation, our proposition is that the transition towards sociobioeconomy fundamentally depends on the quality of the governance that structures these initiatives. We investigate the following question: how do institutional and organizational governance elements support sociotechnical transitions to sociobioeconomy and what specific mechanisms hold potential for replication in megabiodiverse contexts? For this, we analyze four experiences that reconcile conservation and income generation for Amazonian populations.

This analysis is especially relevant in light of the complexity of the value chains in socio-bioeconomics, reinforcing that the construction of new trajectories for sustainable development in the Amazon requires substantive transformations that go beyond substituting products or practices. It must reach institutional arrangements, governance mechanisms, and methods of value generation and distribution. From a theoretical standpoint, this involves a transition between sociotechnical systems, necessitating interdependent changes in technologies, norms, markets, supply chains, infrastructure, practices, cultural meanings, organizational structures, and governance forms. According to by Geels et al. (2017) and Garrett et al. (2024), transitions emerge through interactions among niches (radical technological or social innovations protected by public policies), the dominant regime, and the broader socio-political landscape.

Through the analysis of experiences in the Amazon context, we aim to identify pathways and challenges for constructing concrete alternatives to the dominant regime of conventional bioeconomy, contributing to the design of effective and replicable transition strategies in megadiverse regions.

## Theoretical Background

### Sustainable Transitions and the Amazonian Context

The sustainable transitions framework proposed by Geels (2011) conceives changes in sociotechnical systems as the result of interactions between three levels: niches, regime, and landscape. The niche represents the protected space (by government or private initiative) where innovations emerge. The regime comprises the set of rules, norms, practices, and established structures that shape (and are shaped by) the dominant system of production and consumption. The landscape corresponds to external structural factors, such as macroeconomic trends, infrastructure, cultural and climatic changes. The transition occurs when the regime, pressured by changes in the landscape and/or innovations in the niches, is replaced or transformed (Geels et al., 2017).

Applied to the Amazonian context, this model helps to understand why even innovative initiatives often remain confined to niches, unable to compete with the dominant regime, often characterized by predatory exploitation of natural resources and low levels of value aggregation. However, the growing pressures in the landscape, such as climate change, decarbonization agreements, and international demands for traceability, place the current regime under tension, creating windows of opportunity for the consolidation of sustainable alternatives to the current regime.

Garrett et al. (2024) argue that establishing new regimes requires interventions to protect sustainable niches, destabilize conventional regimes, and transform institutional frameworks toward socio-environmental justice.

In the case of sociobioeconomics, Avelino et al. (2024) propose that sociotechnical transitions need to consider distributive impacts, the rights of vulnerable groups, and valuing of local knowledge and ways of life, not limited to technological innovations or “green” products. Similarly, Hadfield et al. (2025), in analyzing transitions to sustainability in the Global South, note that trajectories should prioritize community empowerment, respecting their timelines, values, and forms of organization. Thus, institutional arrangements must ensure effective representation of local communities and respect the ways of life that sustain ecosystem conservation, which means recognizing the importance of initiatives based on sociobioeconomic value chains as niches for innovation that could potentially transform the current regime.

### Polycentric Governance

Polycentric governance offers a relevant analytical lens for understanding how sociobioeconomy initiatives emerge, consolidate, and endure in contexts characterized by socioecological complexity, undefined property rights, and historical asymmetries in access to resources and markets. Originally developed by Ostrom (1990, 2010), the polycentric perspective departs from the assumption that no single centralized actor is capable of effectively governing complex socioecological systems. Instead, governance is more likely to be effective when multiple decision-making centers operate with some degree of autonomy, while also being connected through processes of coordination, mutual adjustment, learning, and conflict resolution (Ostrom, 2010).

Based on case studies, Ostrom (2010) identified institutional design principles associated with resilient governance systems, including clearly defined boundaries, locally adapted rules, user participation, monitoring and sanctions, conflict resolution arenas, recognition of community rights, and multilevel coordination. These principles can be grouped into three dimensions: (a) legitimized rules, based on participatory norms creation; (b) monitoring and enforcement:mechanisms ; (c) multilevel governance, capable of adapting rules to dynamic contexts (Bridoux & Stoelhorst, 2022).

In the Amazon context, successful experiences show the presence of polycentric governance forms that respect local knowledge and culture, while also connecting different actors: community associations, social organizations, public bodies, businesses, and Non-Governmental Organizations (NGOs) in cooperative arrangements. Such dynamics align with recent studies in South America, where polycentric arrangements have enabled local communities to exercise autonomy and establish community justice mechanisms, even in contexts marked by state fragility or absence (Chávez-Páez & Beitl, 2025). Local leaders and intermediary organizations often play a key role in mobilizing resources, translating institutional demands, and building alliances.

Recent literature on stakeholder governance and joint value creation provides a bridge between polycentric governance and sustainable transitions, operationalizing Ostrom’s principles in complex socio-economic networks. Stakeholder governance examines how diverse actors, such as local communities, NGOs, and businesses, coordinate their distinct interests to co-create social, environmental, and economic value, rather than merely extracting it (Bridoux & Stoelhorst, 2022). Findings in this field show that joint value creation is maximized when governance relies on relational norms (like trust and fairness) rather than purely transactional or power-based interactions (Bridoux & Stoelhorst, 2022; Dias et al., 2025).

This approach is useful for this study because, in the Amazonian context, conservation and transition efforts often involve market-oriented niches where external partners interact with traditional communities. This is implicitly embedded within ‘Legitimized rules’ and it is directly operationalized by the variable ‘Value distribution mechanisms’. This ensures that the analytical framework assesses whether the benefits generated by the sociobioeconomic transition are equitably shared, preventing elite capture and maintaining the long-term resilience of the polycentric arrangement. Ultimately, this focus on equitable value sharing helps to address the ‘black box of power’ in polycentric environmental governance (Morrison et al., 2019), uncovering unequal capacities and imbalances among actors that directly influence the goals and outcomes of such multi-actor arrangements.

### Analytical Framework for Transition to Sociobioeconomics in the Amazon

The proposed analysis is based on two complementary theoretical dimensions: sustainable transitions and polycentric governance. These theories, together, allow us to understand how governance arrangements in niches are fundamental to supporting the necessary changes for enabling transitions between sociotechnical systems, especially in contexts where there is no support and protection to allow innovations in niches to develop and potentially transform or replace the existing regime (Geels, 2011; Geels et al., 2017).

From this articulation, we propose a theoretical matrix (Table 1) that organizes the main dimensions (three), foundations, variables, and elements of analysis of the investigated cases, providing a structured framework to identify the institutional and organizational elements that have been decisive for enabling the transition to the sociobioeconomy of initiatives in Amazonian contexts.

**Table 1:** Dimensions of analysis for the transition to sociobioeconomy

Dimension	Theoretical Foundation	Variables and Elements for Case Analysis
Sustainable Transitions	Geels (2011); Garrett et al. (2024); Avelino et al. (2024); Hadfield et al. (2025)	• Context and status quo: landscape (economic, political, environmental); • Characterization of the initiative (Niche); • Degree of innovation and institutional disruption; • Barriers or facilitators for the expansion of the niche;
Polycentric Governance	Ostrom (1990, 2010); Bridoux & Stoelhorst (2022); Morrison et al. (2019); Chávez-Páez & Beitl (2025).	• Mapping multiple decision-making centers (Stakeholder Governance); • Legitimized rules and community enforcement (Conflict resolution traditional knowledge and community autonomy); • Joint value creation and distribution mechanisms (power balance among stakeholders).
Signs of transition and observed outcomes	Geels (2011); Geels et al. 2017); Garrett et al. (2024);	• Diffusion and consolidation of the initiative; • Environmental, economic, social, and governance-related outcomes; • Consumer recognition and market valorization.

Source: The authors

The framework does not assume that all cases will display the same degree of consolidation or the same type of evidence. Rather, it provides a common analytical structure to compare initiatives at different stages of maturity and with distinct empirical manifestations. This is particularly important in the Amazonia, where sociobioeconomy trajectories are shaped by territorial specificities, uneven institutional capacities, and different forms of interaction between local actors and broader governance.

The three dimensions—sustainable transitions, polycentric governance and signs of transition and observed outcomes—complement each other and are fundamental in Amazonia, where traditional communities have their own ways of using and managing natural resources, rooted in traditional knowledge. Therefore, any initiative to promote a sustainable transition must recognize and be able to adapt to the sociocultural and environmental realities of the territories.

## Method

This study adopts a qualitative and exploratory approach, based on the analysis of multiple case studies. An initial review of literature on Sustainable Transitions and Polycentric Governance concepts was carried out. Next, we adopted case studies as a research method (Eisenhardt, 1989). This combined approach enables the study to delve deeper and gain richer understanding of the unique phenomenon being observed (Miles and Huberman, 1994). Specifically, it seeks to identify viable pathways for building concrete alternatives to the dominant regime of conventional bioeconomy, which can enhance the transition trajectories to sociobioeconomy.

### Data Collection

In line with Eisenhardt et al. (2016) guidelines for the multiple case study method, we chose theoretical sampling. This implies selecting cases in which we can observe the phenomenon of interest. For this purpose, four experiences were selected that represent niches of socioproducer innovation aimed at conservation and inclusion in different states in the Amazon region, based on the following criteria:

i. Linkage to value chains associated with sociobiodiversity.
ii. Action in territories with a significant presence of traditional or indigenous populations.
iii. Existence of practices associated with environmental conservation and local income generation.
iv. Institutional, academic, or community recognition regarding the relevance of the experience.

The cases were selected from research projects conducted by the authors, which have been regularly collaborating since 2016. In 2025, they joined their research efforts in a roundtable at the 16th National Meeting of the Brazilian Society of Ecological Economics (ECOECO), which generated the theoretical grounding for this present manuscript.

The study examines two production-level value chains—sustainably managed pirarucu and agroforestry native cacao—and two transversal governance initiatives (Aíra collective brand and Origens Brasil platform) that connect markets committed to traceability and fair trade. Table 2, below, provides details about the cases and data collection.

**Table 2:** Description of Cases and Data Sources

Case	Description	Data Sources		
		Analyzed Documents (Secondary Sources)	Interviews (Primary Sources)	
			Organization Type and methods	Respondents
Pirarucu (Data collected between 2021-2024)	Mamirauá Sustainable Development Reserve, which created fishing agreements, including a traditional indigenous practice of counting fish in lakes, in state of Amazonas	Reports, fishing agreements, and community internal regulations from associations,support and research organizations	Community associations, NGOs, research institutions, market agents (e.g. buyers, processing, restaurants); participant observation in field studies	36 interviews
Native Cacao (Data collected between 2018-2024)	Production of fine cocoa and bean-to-bar chocolate in degraded areas Transamazônica region (in the state of Pará)		cacao producers and managers of companies and cooperatives; participant observation at fairs and sectoral meetings	22 interviews
Collective Brand Aira (Data collected between 2022-2023)	Creation of a collective brand (Aíra) for *cuias*’ handicraft.	Formal documents of the Association, such as: bylaws, certificates from IPHAN and INPI, reports and minutes.	Association of Riverine Artisans from Santarém (Asarisan) and research institutions	Two workshops with Asarisan members and 3 interviews conducted with members of the Asarisan’s board
Origens Brasil Platform (Data collected between 2018-2024)	Creation of a collaborative platform where companies and communities can trade goods fairly, with guarantee of origin, direct connection among members (no middlemen), and the recognition of communities culture and in keeping the forest alive.	Reports and internal documents from Origens Brasil	Community associations, support institutions, companies, and the network orchestrator	57 interviews

### Data Analysis

Following Eisenhardt (1989) procedures, we employed a within- and cross-case analyses, using content analysis, allowing for categorizing information obtained from texts and generating knowledge about a specific subject. For each case, we used a different interview protocol, as the initial research project from each researcher had its own research goals and created its own analytical categories. Nevertheless, all of them used an inductive approach to understand the organizations’ governance, how they were initially structured, and how they evolved over time. This was the common point that provoked the discussion and comparison of cases in the roundtable at ECOECO, which consequently was analyzed through the lens of sustainable transitions and polycentric governance.

**Fig. 1:**
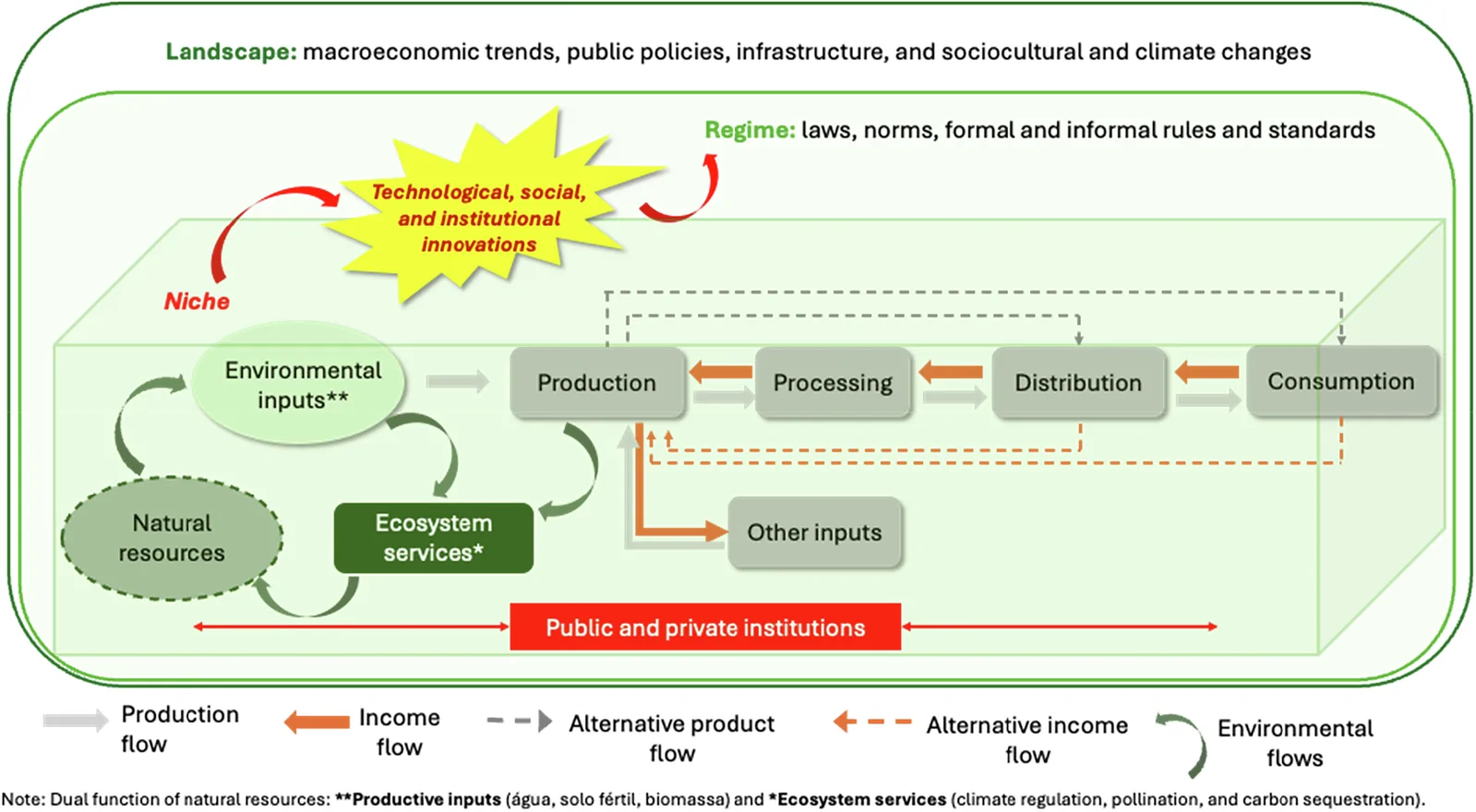
Value chain analysis model. Source: Adapted from Saes et al. (2023)

For the analysis of the value chains associated with each initiative, we propose a conceptual model that recognizes the dual function of natural resources: on one side, as productive inputs (water, fertile soil, biomass); and on the other, as providers of ecosystem services (climate regulation, pollination, carbon sequestration). The structure also encompasses the main links of the chain, connecting environmental inputs, ecosystem services, production, processing, distribution, and consumption, while incorporating the multi-level perspective of sociotechnical transitions (niche, regime, and landscape). Additionally, it considers the different flows of the production process, income, and their respective alternative flows, as well as the generation of environmental benefits. Figure 1 summarizes this analysis model.

This model, combined with the theoretical matrix (Table 1), allows for the identification of the position of the niche initiative and the mapping of implications on the productive links by identifying how the sharing of information and value occurs and the ways in which nature is integrated into the production process, both as an input and as an ecological asset.

Although the analytical framework adopted here draws on value chain and sociotechnical transitions approaches, we recognize that representing nature as a productive input and a provider of ecosystem services is a necessary analytical simplification. For many Amazonian Indigenous and traditional communities, nature is not an external resource, but a constitutive part of territory, identity, and ways of life (Merçon, 2025). Therefore, the notion of sociobioeconomy adopted in this article also requires acknowledging these relational understandings of human–nature interdependence, even if they are not fully encompassed by the analytical categories mobilized here.

## Results

### Sustainable Management of Pirarucu in the Amazon

Sustainable pirarucu management demonstrates how niche strategies shaped by innovative local arrangements can solidify as alternatives to the dominant regime in the Amazon, despite structural and political barriers.

#### Sustainable Transitions: Niche Strategy in the Producer Segment

Pirarucu is one of the largest freshwater species in the world, reaching up to three meters in length and weighing 200 kilograms. It is central to the diet of Amazonian populations and riverside culture, being present in markets, festivals, and culinary traditions (Castello & Stewart, 2010).

The history of pirarucu management is linked to the collapse of pirarucu stocks due to predatory exploitation throughout the 20th century, which led to its inclusion in the list of endangered species (Gonçalves et al., 2018). In the traditional value chain model, the links were organized around unregulated capture, a strong dependence on intermediaries, and a lack of monitoring and stock regeneration mechanisms. The income was primarily appropriated outside of local communities, while the environmental costs fell on the territories. (Castello et al., 2009).

Three IBAMA Normative Instructions (NI) were decisive for pirarucu management. NI No. 29/2002 recognized local fishing agreements. NI No. 34/2004 consolidated closed season (defeso) norms for authorized areas, excluding them from the general prohibition. NI No. 01/2005 introduced individual fish tagging to distinguish managed from illegal catches. This supportive legislation resulted from community governance backed by organizations like IDSM (Mamirauá Institute for Sustainable Development), which systematized practices and expanded regulatory acceptance (Castello, 2004).

The main innovation was the creation of Fishing Agreements (FAs) that integrate traditional knowledge (fish counting), with mechanisms of ‘community justice’ (Chávez-Páez & Beitl, 2025). The counting methodology, developed by IDSM researchers with the local fishers, identifies pirarucu populations by observing the characteristic surfacing behavior known as the *“boiada” (in Portuguese)*. It forms the technical basis of the governance system, as it determines the annual harvest quota authorized by the IBAMA, which limits catches up to 30% of the adult population over 1.5 m (Castello, 2004). Each captured fish receives an individual tag recording its origin, size, sex, and date of catch, ensuring traceability and legal compliance throughout the value chain (Gonçalves, 2024)

This arrangement allows the community to exercise autonomous monitoring and enforcement. The FAs combine traditional and scientific knowledge, strengthening the legitimacy of the process and reconfiguring the value chain on sustainable bases. Legitimized rules include formal elements, such as fishing quotas, minimum sizes, annual closed seasons (*defeso*), as well as informal norms that sustain social cohesion and collective action: the organization of fishing in cooperative groups, the equitable distribution of proceeds among management group members after deducting operational and surveillance costs, and the principle that quota size is proportional to the monitored stock, creating a powerful collective incentive for conservation. As one community leader articulated:

“The pirarucu quota has great meaning for the community to protect [the resource], because the harvest quota they receive from IBAMA is proportional to the stock. So if they don’t protect it, there’s no stock. And if the stock is smaller, the revenue will be smaller. So this equation is something the community values greatly.” (Group Interview [Producers Associations], 2021).

In this new configuration, the value chain for pirarucu now encompasses: (i) zoning, monitoring, and counting of pirarucu; (ii) fishing the authorized quota; (iii) local processing, highlighting refrigerated facilities and drying and salting techniques; and (iv) organized marketing, which reduced dependence on intermediaries and expanded market diversification, including institutional channels, restaurants, chefs, and leather sales. Despite the advancements, significant challenges remain, such as the high logistical costs of monitoring (around 30% of the total production cost), reliance on regulatory bureaucracies, local processing, the persistence of illegal fishing, and barriers to entry into differentiated markets that recognize the added value from management practices undertaken by communities (Saes et al., 2023).

#### Polycentric Governance of Pirarucu Management

The niche strategy was supported by a polycentric arrangement, as proposed by Ostrom (2010). The governance of pirarucu management is characterized by the articulation between different scales and actors, bringing together multiple decision-making centers: local communities, community associations, fishermen’s colonies, technical support organizations, partner NGOs, environmental agencies (including federal, state and municipal agencies), buyers, institutional markets, tanneries, and restaurant owners from various regions of Brazil, especially from the southeast.

Coordination occurs through deliberation forums, where socially legitimized rules (quotas, minimum sizes, closed fishing seasons) are monitored by the community and enforced through a combination of local mechanisms and legal support.

At the community level, annual general assemblies constitute the central deliberative arena where rules are discussed, decisions about investments are made collectively, and quotas are established based on the participatory fish count, as an interviewee said:

We hold a general assembly with them [community fishers], then an investment definition assembly, where everyone voices their ideas. We gather everything afterwards, draft it, read back all that was discussed and deliberated, approve it, everyone signs, and the process moves forward together to justify what was deliberated. […] And the great thing is that you are giving them autonomy to decide. It is them who are deciding certain things. They feel incredibly important.” (FAS [NGO], 2022).

In this case, community assemblies also serve as spaces for conflict resolution and institutional adaptation, ensuring legitimacy and inclusion through the broad participation of riverside families in all stages of the process.

The enforcement system operates through an hybrid arrangement that compensates for the inherent limitations of state oversight in remote Amazonian territories (Chávez-Páez & Beitl, 2025). Formal enforcement is carried out jointly by Chico Mendes Institute for Biodiversity Conservation (ICMBio) and IBAMA, whose operational capacity is structurally limited.

This institutional gap is filled by Voluntary Environmental Agents from the communities, who monitor lakes during critical periods (December–June), prevent illegal entry, and report infractions. While formal sanctions remain under IBAMA and ICMBio authority, community monitoring creates a first line of defense rooted in peer accountability and shared economic interests.

The effectiveness of this hybrid enforcement system is reflected in the reduction of illegal fishing. As the extractivist reserve manager said:

“When we started the pirarucu management here, in a single enforcement trip we would seize one ton, one and a half tons of illegal pirarucu. Do you know what the result of the last three enforcement trips was? Zero kilos of pirarucu. […] No one is foolish enough to go kill a pirarucu clandestinely in their lake and sell it for a pittance, if through management they earn three times more from it.” (ICMBio [Federal Environmental Agency], 2024).

This observation aligns with the stakeholder governance theory (Bridoux & Stoelhorst, 2022), when the economic benefits of the legitimate arrangement are distributed equitably and visibly, voluntary compliance becomes the dominant strategy, reducing the need for coercive enforcement.

This multi-arena, multi-level enforcement system, including encompassing assemblies, community watchmen, partner NGOs, state environmental agencies, and even federal police when organized crime intersects with illegal fishing, exemplifies the polycentric logic described by Ostrom (1990), wherein no single center holds absolute authority, but generates adaptive and resilient institutional outcomes.

The commercialization through community associations has raised the prices paid to fishermen, allowing for collective investments, and has included managed pirarucu in institutional and differentiated markets (like Brazilian National School Feeding Program—PNAE), reducing asymmetry in relation to middlemen, albeit still incipient for some cases (Morrison et al., 2019; Chávez-Páez & Beitl, 2025). In addition to economic gains, management has strengthened social cohesion and cultural recognition (Campos-Silva & Peres, 2016; Freitas et al., 2020). The pirarucu is not only an economic resource but a species deeply embedded in ribeirinho identity — as one community member stated, *“much more than an economic alternative, it connects to the culture of this population”* (IDSM [Intermediary technical-scientific organization], 2022). The creation of collective brands (such as “Pirarucu de Manejo de Mamirauá,” now protected by a Geographical Indication) and partnerships with high-end gastronomy have given this cultural identity visibility in national and international markets.

#### Results and Signs of Transition to the Regime

Since the first initiative implemented in 1999, sustainable practices have increased stocks in managed lakes by approximately 620%, while non-managed areas continued to decline (IDSM, 2025; Campos-Silva & Peres, 2016). The activity has become a major source of income for the communities, promoting food security, reducing socioeconomic vulnerability, and enhancing collective self-esteem (Saes et al., 2023).

Sustainable pirarucu management emerged as a niche innovation that evolved into a replicable social technology, supporting a broader institutional change. The data show that there are currently 48 agreements covering 25 municipalities, benefiting around 415 communities and more than 8,000 families (SEMA, n.d.). Recently, the Sociobiodiversity Payment for Environmental Services Program for community-based pirarucu management was approved through Ordinance MMA/MDA n°. 1.645 (MMA & MDA, 2026).

The sustainable pirarucu management in Amazonas exemplifies a transition toward sociobioeconomy by replacing predatory practices, creating new local institutions, and gaining regulatory recognition. In terms of governance, it shows how multiple decision-making centers articulate inclusively and adaptively, combining community monitoring, social legitimacy, and multiscale coordination.

These transformations are also reflected in the perceptions of community members, who associate pirarucu management not only with income generation, but also with greater community organization, education, and collective unity. As one interviewee stated,

“We know that it has completely changed everyone’s life. […]We see the Community more organized, people living in a more organized way. Pirarucu also walks together with education for the Community, walks together with the unity of the Community, walks together with partnerships, but we still hope that it will have a fair price” (AMAB [Community Association], 2024).

The results, including ecological recovery, social inclusion, value distribution, and cultural appreciation, highlight the potential for fair, resilient, and inclusive trajectories when science and traditional knowledge converge. Among the barriers, notable challenges include the costs of surveillance, the persistence of illegal fishing, and logistical difficulties (Freitas et al., 2020).

### The Production of Fine Cocoa and Chocolate in Transamazônica

The production of fine cocoa and chocolate in the Transamazônica (in the state of Pará) involves producers, cooperatives, NGOs, research institutions, and universities. This initiative has not only enabled the regeneration of degraded areas but also brought significant economic and social gains. In particular, it contributes to a potential break from the historically developed model—focused on commodity production without socio-environmental concern, heavy reliance on middlemen, vulnerability to market fluctuations, and low participation of women in the bioeconomy. In this sense, the cocoa from Transamazônica can be recognized as a movement of partial reconfiguration of the traditional regime, coexisting in a relationship that is not only confrontational but also complementary and synergistic.

#### Sustainable Transitions: A Niche Strategy in the Fine Cocoa and Chocolate Segment

The cultivation of cocoa in Transamazônica began in the 1970s, driven by the government’s agricultural colonization policies. This cultivation developed amidst limited market diversification and low technical qualification, creating a favorable context for the dominance of middlemen. Aside from generating significant asymmetries between the processes of value creation and distribution, this configuration helped perpetuate a trend for decades in which profits were contracted outside the region, while the social and environmental costs remained within local communities.

From the 2000s onwards, pressures at the landscape level—such as the appreciation of premium cocoa, international demands for traceability, and advancements in Amazon conservation policies—created opportunities for a new niche to emerge: the production of fine cocoa and bean-to-bar chocolate (Folhes & Serra, 2023). In this context, cooperatives like COOPATRANS (the owner of the Cacauway brand) began to structure and operate as disseminators of controlled fermentation techniques, standardized drying, and the processing of cocoa beans, as well as producing fine chocolates and various other high-quality cocoa-based by-products (Fernandes, Comini & Rodrigues, 2022). In light of Geels’ (2011), it can therefore be inferred that this fine cocoa and chocolate niche began to function as a protected space that houses socio-environmental and technological experiments, offering alternatives to the traditional regime characterized by cultivation solely aimed at commodity markets.

This new fine cocoa niche combines technical, organizational, and institutional innovations. At the technical and organizational levels, this shift began in the late 2000s through the combined action of producers and public support, like cooperatives improving local value addition, and state programs such as FUNCACAU and PROCACAU promoting technical assistance, agroforestry systems (SAF’s), quality improvement, and processing (Venturieri et al. 2022). At the institutional level, advancements in bioeconomy policies, technological innovation, and private investments increased access to differentiated markets and international certifications. Together, these innovations supported cooperatives, improved quality, reduced dependence on middlemen, and expanded access to differentiated markets (Gontijo, 2020; Folhes & Serra, 2023).

Following these innovations, small cooperative and family-based enterprise began to adopt value-adding strategies through the production of bean-to-bar chocolates and products like cocoa powder, pastes, and creams which directly utilize the beans produced in the region (Carbone & de Castro, 2026). This shift became more pronounced in the late 2010s, when organizations such as CEPLAC, UFPA and SEBRAE started to help the creation of local capacity for processing and value retention (Nunes & Bastos, 2018). It is worth noting that the region has always been a stage for historical conflicts, particularly concerning illegal logging and the Belo Monte hydroelectric power plant, built from 2011.

Despite the advancements, the consolidation of the fine cocoa and chocolate niche faces structural challenges in Transamazônica. Among the main barriers are: (i) poor logistics (exacerbated by the bad road conditions) and high transportation costs; (ii) limited access to credit, agricultural insurance, and specialized technical assistance, despite efforts from universities and federal institutions; (iii) the high costs of international certifications and traceability requirements, which distance some producers from the main fine chocolate markets.

#### Polycentric Governance in the Fine Cocoa and Chocolate Sector of the Transamazônica

The emergence of the niche for fine cocoa and chocolate in the Transamazônican is directly associated with a polycentric governance arrangement, where multiple decision-making centers operate interdependently and in a coordinated manner (Ostrom 1990; 2010). In addition to government agencies, local cooperatives have also played a strategic role in advancing the sector, exemplified by COOPATRANS (responsible for the Cacauway brand) and CEPOTX (Central of Organic Production Cooperatives of Transamazônican and Xingu). By collaborating with public agencies and research institutes—such as EMBRAPA, CEPLAC, the Federal University of Pará (UFPA)—these actors have contributed to the formation of an institutional network responsible for implementing good agricultural practices, advancing certifications, and significantly increasing local processing, creating conditions to insert Transamazônica cocoa into higher value-added markets, a long-held dream now supported by a previous collective achievement: the creation of a funding mechanism for cocoa farming, the Cocoa Development Fund in Pará (Funcacau). In line with Garrett et al. (2024), this case not only approaches adaptive governance, capable of handling institutional and market changes, but has also induced these changes throughout its trajectory.

The governance of fine cocoa in the region is based on the collective legitimacy of production and marketing rules, built through participatory processes (Carbone & de Castro, 2026) and supported by Ostrom’s (1990) principles of institutional design. Assemblies, forums, and councils—such as those linked to the Cocoa Route, an initiative supported since 2018 by the Ministry of Integration and Regional Development (MIDR)—help jointly define strategic guidelines and actions for advancing cocoa producers. Additionally, they serve as spaces for exchanging knowledge and practices related to handling and processing—fermentation, drying, cocoa bean quality, and traceability, among others. Initiatives like organic certification and good handling practices protocols have been incorporated as fundamental tools for accessing premium markets. Furthermore, public policies have strengthened these initiatives by encouraging regenerative practices, the recovery of degraded areas, and an increasing promotion of agroforestry systems (SAFs) in cocoa cultivation.

Regarding the monitoring mechanisms of polycentric governance, there is a combination of collective control and external oversight. Cooperatives, associations, and other support organizations, such as universities and NGOs, provide training and self-inspection processes to monitor handling practices, while buyers work with certifications that attest to and pay an additional value for standards of differentiated markets.

In summary, the fine cocoa and chocolate sector of the Transamazônica demonstrates that strengthening the polycentric model can indeed elevate value capture among producers in the region, who have managed to internalize processing stages and diversify marketing channels, generating greater value addition and empowering producers who traditionally received much lower returns from the sale of bulk cocoa to a very limited group of companies, which control around 90% of country’s cocoa bean market (Coslovsky, 2023) and treat the fruit as a commodity, using it primarily for the production of low-quality chocolates and selling them in the domestic market.

#### Results and Signs of Transition to the Regime

The results indicate that, despite the difficulties, the Transamazônica region is becoming established as a laboratory for sociotechnical innovation in the fine cocoa and chocolate supply chain, promoting a gradual reconfiguration of the local production system, marked by innovations, environmental conservation, and connections to the global bioeconomy agenda (Avelino et al., 2024). The strengthening of SAFs and management practices has helped reduce pressure on forest, promoting the regeneration and increasing biodiversity through native species. At the same time, techniques and instruments for fermentation, drying of beans, and processing have led to improved cocoa quality, the achievement of national and international awards, and access to new markets. In summary, there has been a progressive shift from the traditional model, based on selling bulk cocoa to intermediaries, to a more sustainable regime. Based on the analyses, it is observed that the formation of a robust polycentric governance was crucial for this transition. In particular, when considering the journey of local cooperatives and the collaborations with support organizations and universities. Together, these actors have created a multiscale network of technical and institutional support, promoting central decision-making forums and arenas to establish collectively legitimized rules, define quality protocols, and strengthen integration among producers, certifiers, and buyers.

Additionally, this collective coordination has enhanced the institutional capacity for adaptation and response to the demands of markets and financiers, creating new opportunities for small and medium producers. Among the most significant results is the increased ability of local actors to capture value, either through improvements in the processing methods or by expanding their portfolios and entering new markets. The most evident achievement of this resides in the cooperatives that did not previously exist and are now consolidating their bean-to-bar chocolates and creating their own shops, allowing for income diversification and reducing the historical dependence on middlemen. Besides signaling that transitions are possible, as long as polycentric networks are strengthened, this case attests to the decisive role of public policies aimed at economic development and environmental conservation in regions historically characterized by conflicts and environmental neglect in the Amazon.

### The Collective Brand Aíra

The *cuias’* handicraft is an activity within the sociobioeconomy of the Amazon that involves traditional knowledge, cultural practices, the use of natural resources, and income generation for riverside communities, especially in the Lower Amazon region of the Pará State. In 2014, the Asarisan registered the first collective brand in the Pará State, Aíra, an innovation at the niche level with great potential to promote transformations within the regime.

#### Sustainable Transitions: Niche Strategy in the Cuias’ Handicraft from Lower Amazon

*Cuias* are objects produced from the *cuieira’s* fruit (*Crescentia cujete*) by Indigenous people of the Brazilian Amazon for at least three centuries. It is possible to observe the presence of *cuias* in all Indigenous houses, used as bowls and cups. Over the centuries, the *cuias’* handicraft spread to riverside communities throughout the Amazon, becoming strongly established in the Lower Amazon region, due, among other factors, to the abundance of *cuieira* tree (Carvalho, 2011). This movement allowed *cuias* to be incorporated into the local food culture to such an extent that, in contemporary times, they are mainly used locally to serve regional food.

Despite all the traditional knowledge associated with the *cuias’* handicraft, the sociocultural importance of this process, its use and symbolic value, as well as its potential to generate income for riverside communities and empower women, for many years, artisans sold their handicrafts to middlemen, who paid them very low prices and resold the products to handicraft stores.

However, in the late 1990s, two changes in the landscape contributed to the emergence of innovation and the subsequent creation of a niche. The first was the Industrial Property Law No. 9279 in 1996, which, among other things, regulated collective distinctive signs, such as geographical indications (IG) and collective brands (MC) (Miléo & Sampaio Júnior, 2012). The second change began in 1998 with the first surveys conducted by the National Center for Folklore and Popular Culture (CNFCP), linked to the National Institute of Historical and Artistic Heritage (IPHAN), aimed at valuing the *cuias’* handicraft.

The actions of the CNFCP, along with regional groups dedicated to valuing local culture, spurred a movement to rescue the traditional *cuias’* handicraft and seek mechanisms for recognition and appreciation of the products. As a result of this movement, Asarisan was created in 2003, bringing together a group of artisans willing to seek mechanisms to value the *cuias’* handicraft and empower women. As one interviewee stated, “*We want to give visibility to our work and we were encouraged by several groups such as SEBRAE and CNFCP to unite collectively” (Member of the Asarisan board, 2022)*.

From the creation of Asarisan, the movement to form a niche that could drive a sustainable transition intensified, and in 2014, Asarisan registered the collective brand Aíra, with technical support from a project at the Federal University of Western Pará (Ufopa). In 2015, IPHAN granted registration of Brazil’s Cultural Heritage designation to the traditional handicraft of making *cuias* from Lower Amazon.

According to the theory of sustainable transitions (Geels, 2011; Garrett et al., 2024), it can be inferred that the creation of the collective brand Aíra was a niche strategy with great potential to promote changes in the regime. Indeed, some results indicate that a transition process has been initiated, as will be presented in the following results.

#### Polycentric Governance: in the Cuias’ Handicraft from Lower Amazon

The creation of the collective brand was the result of the action of various organizations that, with their specific expertise, supported Asarisan and fostered the emergence of this niche. From valuing handicrafts initiated by the CNFCP, to the support for participation in fairs and mediation for access to new markets provided by SEBRAE and Artesol, culminating in the Legal Advisory of the project “Distinctive Signs and Access to Markets” from Ufopa (Miléo & Sampaio Júnior, 2012), all partners were fundamental in the process. As one interviewee stated, “*We received a lot of support in the beginning, from various organizations, and we took many courses that helped us a lot” (Member of the Asarisan board, 2022)*.

However, although these partners provide support, the management of the collective brand is carried out by Asarisan, which, in addition to commercial and financial management, must ensure compliance with the Brand Use Regulation registered with the National Institute of Industrial Property (INPI), since 2014. Among other things, the Regulation establishes quality criteria, sustainable ways of using natural resources, design standards, rights of brand use, and the organization of production in hubs (groups of communities).

The extensive set of responsibilities of the Association regarding management is identified by the artisans as one of the difficulties in consolidating the results of the collective brand. The lack of support to deal with bureaucratic issues regarding brand use, along with business environment challenges not adapted for community enterprises, makes the Association dependent on partners. Instances like the *Paraense* Forum for Geographical Indications and Collective Brands created in 2016 can and should function as an “orchestrator” that provides support not only for Aíra but for all IGs and MCs in the State (Pará, 2024), effectively establishing a polycentric governance in the mold of Ostrom (1990; 2010) that truly strengthens the niche and allows it to drive transformation in the regime.

However, the Aíra case also reveals the limits of polycentricity when stakeholder governance is not fully horizontal. The artisans’ dependence on institutional partners for bureaucratic management suggests that power imbalances still persist within the niche. As Morrison et al. (2019) argue, uncovering these ‘hidden’ power asymmetries is fundamental; in the case of the Aíra brand, the lack of full administrative autonomy limits the artisans’ capacity for joint value creation, even when the symbolic value of the product is high.

#### Results and Signs of Transition to the Regime

Two results emerge as most significant with the creation of the collective brand Aíra. First, access to new markets, such as tourist centers, handicraft fairs throughout Brazil, and online sales through the brand’s own website. The second result is the added value to the product, with the expansion of the market and the portfolio of items produced from the *cuieira’s* fruit, such as vases, fruit bowls, cups, and *maracas*.

The direct consequence of these results was the elimination of the middleman and, consequently, the guarantee of better selling prices for the handicrafts. While the middleman pays US$0.29 per painted *cuias*, on the brand’s website the prices of the *cuias* range from US$1.56 to US$3.92, meaning the price on the brand’s own website is at least 5 times higher than what the middleman pays. This price increase, in addition to generating income, demonstrates that the symbolic value embedded in *cuias’* handicrafts could be represented in monetary gains for the artisans, guaranteeing not only recognition but also the appreciation of traditional knowledge.

By promoting changes in marketing practices and community strengthening, the niche strategy through the collective brand impacts socio-economic, cultural, and environmental dimensions, indicating signs of an ongoing just regime transition, according to Avelino et al. (2024). However, some elements of the landscape limit the potential of the niche in promoting the transition. Among these limiting elements, several can be highlighted: a) structural factors, such as the difficulty of transportation and communication for riverside communities; b) lack of government support; c) lack of articulation with a local network of traders in the tourism sector; and d) a business environment not adapted for community enterprises, with standardized agreements, without flexibility. Therefore, overcoming the limiting elements of the landscape becomes imperative for the collective brand (niche) to develop its full potential for transforming the regime in order to consolidate a just and inclusive sociobioeconomy. Proposing as a first step in this direction the strengthening of the State Forum for IGs and MCs to act as an “orchestrator” and promoter of changes in the landscape.

### The Origens Brasil Platform

Origens Brasil is a platform orchestrated by Imaflora (an NGO) and composed of 206 stakeholders (39 companies, 79 communities, and 88 NGOs), operating in conservation areas of the Amazon basin, covering 62 million hectares (Origens Brasil, 2024).

The platform aims to promote ethical businesses, connecting companies directly with traditional communities to negotiate and trade inputs as well as co-create products.

#### Sustainable Transitions: Strategy for Empowering Niches

This initiative was a response to significant external pressures—illegal deforestation, mining, and cattle ranching—that threatened these communities. Initial diagnoses revealed some challenges: remote communities with limited market access, undervalued products often sold via middlemen, and scarce technical support, all exacerbated by intense illegal activities.

The project’s central objective was to establish a resilient system that upheld conservation rules and ethical trade principles, respected traditional knowledge, and delivered added value for participating companies. Origens Brasil was built upon established key pillars: provenance through traceability, transparency in commercial relationships, and defined rules for ethical and balanced trade. These principles are aligned with the interventions proposed by Garrett et al. (2024), to promote sustainable transitions and were critical in strengthening operational niches and disrupting conventional, exploitative regimes, such as those relying on middlemen and minimal value capture by local communities.

In terms of ethical and balanced trade rules, it is stipulated that each party has equal power in negotiations. To ensure such parity, both Imaflora and supporting institutions monitor and evaluate commercial partnerships between companies and communities based on four pillars: (i) transparency and open, equitable dialogue between the parties, (ii) guarantee of fair prices, (iii) commercial relationships that respect the traditional way of life of communities, and (iv) commercial relations formalized in contracts or cooperation agreements. Thus, the sociotechnical transition promoted by Origens Brasil platform ensures that values can be appropriated distributively among the parties, that the rights of more vulnerable groups are respected, and that local knowledge and ways of life are valued (Avelino et al., 2024).

#### Polycentric Governance of the Origens Brasil Network

The governance of the network features elements of both centralization and decentralization. On the one hand, Imaflora is responsible for ensuring that the entry and permanence rules (e.g., the network’s Ethical Trade Guide) are followed by all members. On the other hand, the rules are discussed, negotiated, and established collectively among the platform’s members. Moreover, Imaflora does not mediate negotiations, which are conducted directly between producers and companies. All conflicts and tensions that may arise are resolved through open and direct dialogue between the parties. This model of polycentric governance clearly offers greater legitimacy, as various actors are involved in establishing the rules and mechanisms for monitoring and conflict resolution (Ostrom, 1990).

Imaflora acts as the orchestrator of the platform, initially aiming to implement the necessary organizational changes for each group of stakeholders, and more recently focusing on enhancing joint solutions among its members. For this enhancement to take place, Origens Brasil has been structured over the years with a multi-level governance approach. Initially, Territorial Committees were created, where communities share their experiences in collection, production, value addition, and commercial relations. A Committee for Companies was also established, where, similarly, companies share experiences regarding the existence and applicability of certain inputs, learn how other companies are adapting their commercial relations to work with communities, and how to overcome the logistical challenges of the Amazon region. Finally, a face-to-face event called “Encontrão” was created, where all stakeholders connect, share experiences, co-create products, and establish new commercial relations.

Ultimately, the Origens Brasil platform functions as a sophisticated mechanism for stakeholder governance (Morrison et al., 2019) in Amazonian trade. By formalizing ethical trade rules and providing equal power in negotiations, the network ensures that joint value creation is not captured only by stronger commercial actors. This balanced polycentric arrangement allows traditional communities to maintain their autonomy and way of life while successfully interacting with modern market landscapes.

#### Results and Signs of Transition to the Regime

The main outcome achieved by the platform is the direct connection between chain links, and the rules of ethical trade have led to greater value appropriation, especially for the most vulnerable groups. Furthermore, this closeness has also allowed for the development of various innovations, such as the creation of mini power plants, co-creation of products, and the establishment and marketing of the communities’ own brands.

There are also four other important indicators reported by community members through interviews and analysed in secondary sources (reports). The first is the return of community members to their villages after seeking opportunities in cities. This is particularly notable among the youth. There has also been a reduction in the outmigration of people from the communities.

“In some communities we see the return of some people who had gone to the cities in search of something better, but after the arrival of Origens Brasil, they decided to return because the community’s income increased.” (Imaflora manager)

“One of the things that greatly encouraged it, through this initiative to improve the market and products, was the stimulus it provided to the youth. […] Before this, there was a very high rate of exodus from the village; this source of income was stagnant. So when we sought this alternative with Imaflora, we helped reconnect the young people. Some worked in mining, others went to the city. Another advantage […] is the appreciation of the elders, because you’ve valued our own production chain, encouraging young people to want to learn about it, and on the other hand, keeping the community within its own territory.” (Community Leader)

The second indicator is a better balance between men (53%) and women (47%) participating in income-generating activities (Origens Brasil, 2024). For example, at Encontrão, one community leader mentioned that trading in the past was via barter, and only men took part (women would stay at home), and now they included women in the production activity, which increased their income.

The third indicator relates to improvements in the quality of life. Communities report greater access to equipment and products that previously were harder to obtain due to financial constraints. Also at Encontrão, one community leader shared the experience of building a small facility to process and industrialize some products (mostly Brazilian nuts), and get higher value for the products they sell. The leader reported that their income increased, as well as their working capital and quality of life. For example, they now have access to products such as smartphones and branded clothing.

Finally, it is important to highlight the communities’ perception of a greater appreciation of their culture, knowledge, and their role as guardians of the forest. One of the community leaders mentioned that “it tells the story of our people, of our relationship with nature, so Origens Brasil is the beginning of a journey in which the world will see us as the guardians of the forest.” Another one said, “through traceability, they had an incredible insight, because people aren’t just buying the product, they’re paying for the history, for the conservation.”

As a result of the positive outcomes, the platform scaled up. From 2016 to 2025, there was an increase in the number of people (from 3 to 79), companies (from 5 to 43), territories (from 1 to 6), protected areas (from 11 to 54), and annual commercial transactions (from R$ 520k to R$ 8,334k) (Origens Brasil, 2024).

## Discussion

The analysis of the four cases allows for the identification of recurring patterns and specificities that help explain how different socioeconomic arrangements can advance or are limited in their transition trajectories. Although they operate in distinct productive chains and present varying degrees of organizational complexity, the four experiences share similar structural challenges, such as high operational costs, market asymmetries, and institutional fragilities.

To move beyond a descriptive approach, this discussion employs a cross-case synthesis, comparing how each niche’s internal rules and external scales of governance interact to overcome these barriers. This analysis reveals that the success of transitions depends less on the specific product and more on the robustness of the polycentric arrangements and the transparency of joint value creation.

As suggested by institutional economics, understanding these transitions requires examining shifts in actor interrelations and governance structures. In the pre-transition institutional arrangement, the traditional predatory regime, the system is monocentric and dominated by the middleman, who centralizes information, dictates prices, and captures most of the value, leaving local communities isolated and vulnerable. This configuration concentrates decision-making authority and reinforces asymmetric power relations, limiting collective action and long-term sustainability.

In the post-transition governance arrangement, as illustrated by the Pirarucu and Origens Brasil cases, the system evolves toward a networked polycentric structure. In line with Elinor Ostrom’s framework, decision-making authority becomes distributed across multiple, partially autonomous centers, including community associations, NGOs, and state agencies, interacting through processes of coordination, mutual monitoring, and rule co-creation. This multiplicity of actors creates a system of checks and balances that reduces the risk of value capture by any single stakeholder and enhances the resilience of the arrangement.

From a stakeholder governance perspective, the emergence of an orchestrator (e.g., Imaflora or IDSM) does not imply a return to centralization. Rather, this actor performs a facilitative role, enabling alignment among stakeholders, fostering transparency, and supporting joint value creation. In this sense, the orchestrator operates as a connective node within a polycentric governance arrangement, rather than as a hierarchical authority.

It is notable that the transition toward sustainable practices in these niches is driven by distinct environmental pressures and socio-economic needs. While the Pirarucu case is a recovery effort following the species’ inclusion on the extinction list, Cacao and Origens Brasil focus on meeting global demands for traceability and ethical trade. The *cuias’* handicraft serves as a socio-economic intervention to reduce producer dependence on intermediaries, increase the historically low added value of the handicraft, and promote the appreciation of the traditional knowledge associated with activity.

Innovation across these cases is characterized by the integration of traditional knowledge with modern certification and management systems. Efforts range from the systematization of traditional FAs (Pirarucu) to the development of advanced fermentation technologies (Cacao) to create a collective brand (Aíra) and the creation of dual-function traceability seals (Origens Brasil). Key barriers include difficulties in scaling these initiatives due to high logistical and surveillance costs in the Amazon, limited government support, and significant barriers to international trade.

While analysing polycentric governance, it is noteworthy how it is decentralized and multistakeholder in nature, involving a complex network of actors. Origens Brasil maintains the most extensive network, comprising 206 stakeholders. At a smaller scale, the *cuias* association coordinates 25 artisans. In this setting of polycentric governance, compliance is ensured through diverse instruments, such as FAs, brand use regulations, and ethical trade guides.

In terms of value distribution and to ensure economic sustainability, these cases employ varied value-capture mechanisms. Strategies include accessing institutional markets like the PNAE (Pirarucu), establishing specialized tourist routes (Cacao and *Cuias*), and promoting the co-creation of products (Origens Brasil). Additionally, mechanisms such as distributing orders based on artisan skill or fostering community-owned brands are utilized to ensure fair compensation within the value chain.

The comparative synthesis (Table 3) highlights how factors such as niche type, context, degree of innovation, faced barriers, governance arrangements, and value distribution mechanisms differentially shape the possibilities for consolidating these socioeconomic systems. This comparison allows for the identification of both transversal elements, particularly related to polycentric governance and the legitimacy of rules, as well as important differentiations that influence the pace and depth of the analyzed sociotechnical transitions.

**Table 3:** Synthesis of the case analysis

Dimensions	Experiences			
	Pirarucu	Cacao	*Cuias’* Handicraft	Origens Brasil
Niche	Sustainable management	Cultivation in agroforestry systems	Collective brand	Collaborative platform
Transition				
Context	Prohibition of pirarucu fishing; regulations in management areas.	Increased pressure for sustainable practices and traceability; increased consumption of fine chocolate.	Dependence on intermediaries; low added value.	Guarantee of origin; direct connection from producer to purchasing company; appreciation of communities.
Innovation	Systematization of traditional knowledge for fishing agreements.	Development of methods and technologies for sustainable cacao management and fermentation of beans.	Movement for the appreciation to traditional *cuias’* handicraft in the Lower Amazon	Creation of a traceability seal with dual function.
Barriers	Logistical costs; surveillance costs; illegal fishing; difficulty entering differentiated markets.	Bargaining power of intermediaries; market entry barriers for international markets.	Transport and communication issues; lack of government support; networking challenges; non-adapted business environment.	Scaling the initiative both in volume and geographically; identifying new products and communities.
Governance				
Mapping multiple decision-making centers (organizations)	Community associations, fishers’ colonies, regulatory bodies, research institutions, NGOs, and buyers.	Producers’ associations and cooperatives, multistakeholder participatory forums (Cacao Route), local and national government, NGOs, universities, and large cocoa processors.	Asarisan comprises 25 artisans and has governmental, non-governmental, and research organizations as partners,l	The platform comprises 206 stakeholders, including 43 companies, 79 community organizations, and 88 NGOs (technical and administrative support).
Legitimized rules and community enforcement	Fishing agreements.	Creation of the Geographical Identity of Xingu cacao (still in progress).	Rules established in the Brand Use Regulation.	Territorial committees and companies; evaluation of commercial partnerships; ethical trade guide.
Value distribution mechanisms	Organization of fairs; restaurants; institutional markets; attempt to implement export strategies.	Organization of sectoral events; creation of websites and own stores; creation of a tourist route focused on cocoa and chocolate.	Orders are distributed according to the skill and availability of artisans.	Expansion of spaces for co-creation of products; growth of sales of community-owned brands; organization of platform events.

Source: The authors

## Conclusions

This study aimed to analyze the factors that can enable a transition to the concept of sociobioeconomy, thus contributing to the establishment of a fairer and more sustainable bioeconomy, aligned with the needs of Amazonian communities and territories. Through a dialogical comparison between the 4 studied cases and the literature, we demonstrated that the strengthening of niches is conditioned by different elements: technological, productive, social, and political. Thus, it is possible to perceive that the transition process involves not only the emergence of innovations, markets, and technologies but also reconfigurations in value chains and in society, including the very relationship between nature, economy, and society.

In this sense, the study contributes to repositioning debates on bioeconomy and sociobioeconomy in the Amazon, highlighting that the transition to more inclusive models cannot be based on a purely technological rationale, but also on a relational and political approach. In our four cases, aspects such as local knowledge, the protagonism of the involved communities, and the reconfiguration of power relations allowed for the emergence and expansion of niches.

The importance of collaborative governance and, above all, genuine engagement with the participation of local communities in the development of sociobioeconomy in the Amazon, the study delved into the different elements that facilitate the distribution of power and decision-making in each of the investigated cases, checking that, although they have distinct stages and processes, there is a convergence towards the existence of mechanisms that reconfigure historically constructed power relations in each of the sociobioeconomy chains addressed. By demonstrating how this occurred on a case-by-case basis, the research offers a set of relevant contributions to the literature and to practitioners engaged in this field.

In the theoretical field, it demonstrates how the transition to a sociobioeconomy is conditioned by different temporalities, local contexts, and sociocultural aspects related to the actors involved and the territories in which it develops. Additionally, the study shows how local practices, based on ancestral knowledge (e.g. Pirarucu and *Cuias*) or in the articulation of organizations at different levels and sectors of the economy (e.g. Cocoa and Origens Brasil), are determinants for consolidating fairer and more sustainable practices of production, management, and circulation.

In the practical field, the study reinforces the role of multistakeholder organizational arrangements that effectively integrate grassroots organizations, cooperatives, associations, and a series of other important actors for the advancement of sociobioeconomy in the Amazon. Furthermore, the trajectory of the cases addressed illustrates how territorial governance configures a central element for promoting a polycentric logic, reinforcing that, before aggregating multiple actors in the development of sociobioeconomy chains, there is a pressing need to coordinate efforts that address the demands present in the territories where these chains develop. Especially in the sense of ensuring the valuation of biodiversity, respect for different ways of life, and the appreciation of traditional knowledge.

The study has some limitations that should be acknowledged. First, it is limited by the scope of the method used. Therefore, we suggest, as future research, developing more structured case research, adopting a longitudinal approach, in order to compare the evolution of different set institutional approaches over time. We also would like to suggest strengthening the research, adding more cases in different geographical regions to have a better understanding of transitions to sociobioeconomy in diverse social contexts.

## Data Availability

The data that support the findings of this study are not openly available due to reasons of sensitivity and to protect study participant privacy, but are available from the corresponding author upon reasonable request.
